# Supplementary figures and images for: NELFA-mediated pausing restrains YAP transcription and context-dependent outcomes in breast cancer
Source: Front Oncol. 2026 Apr 22;16:1808415. doi: 10.3389/fonc.2026.1808415 (PMC13143776; doi:10.3389/fonc.2026.1808415)

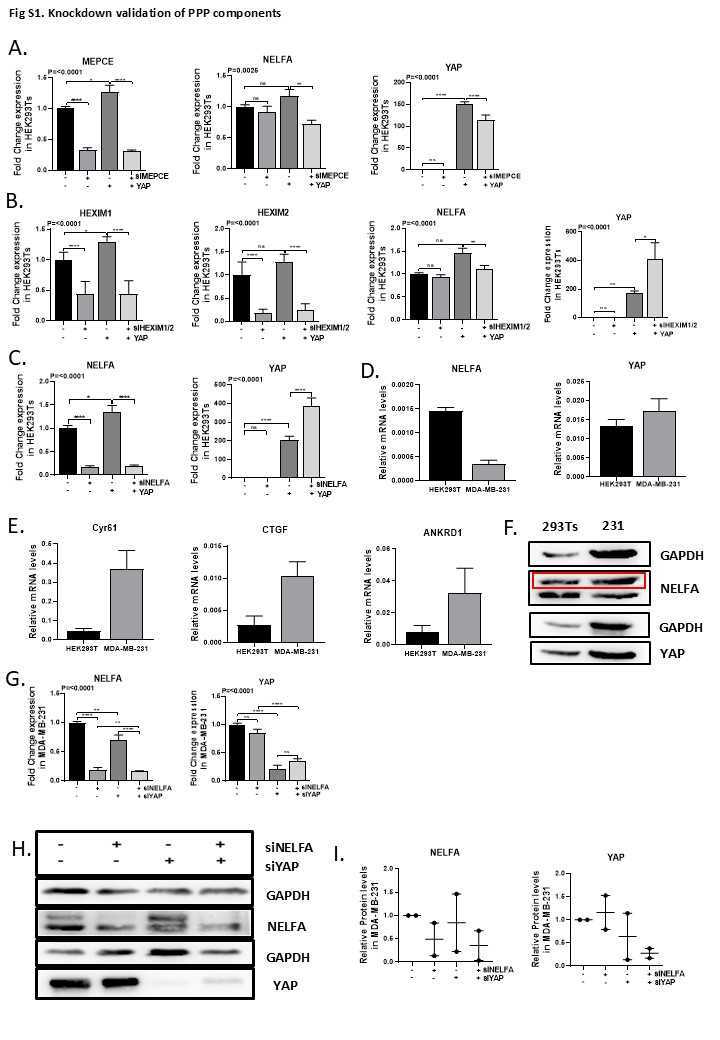

Supplement: Supplementary file 1 [file Image1.jpeg]

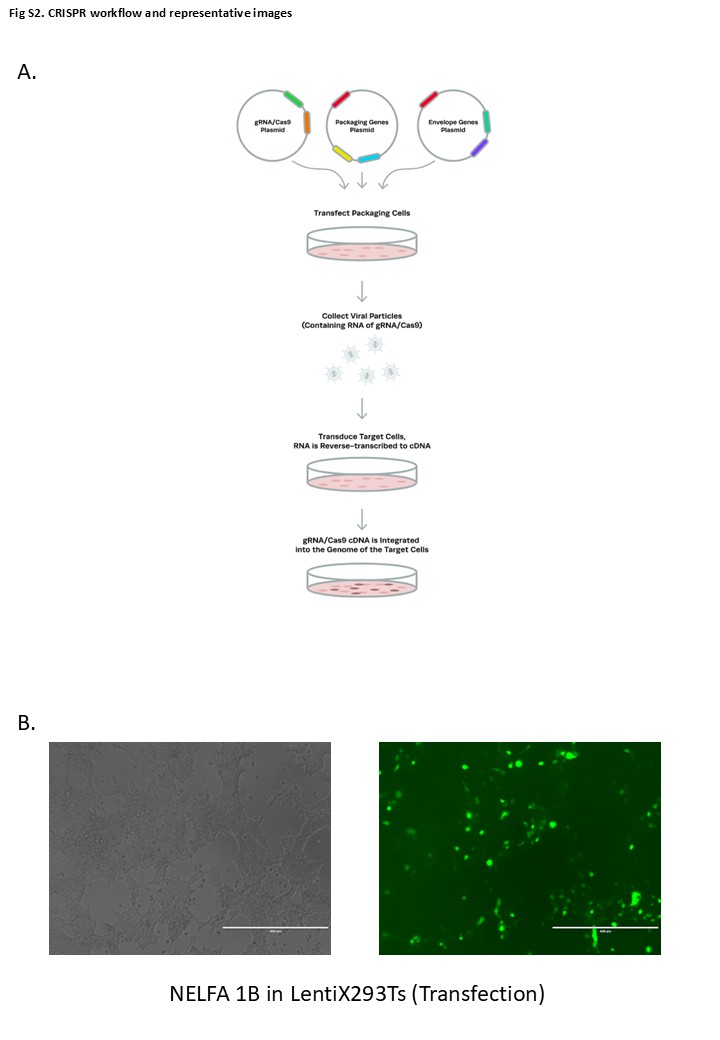

Supplement: Supplementary file 2 [file Image2.jpeg]

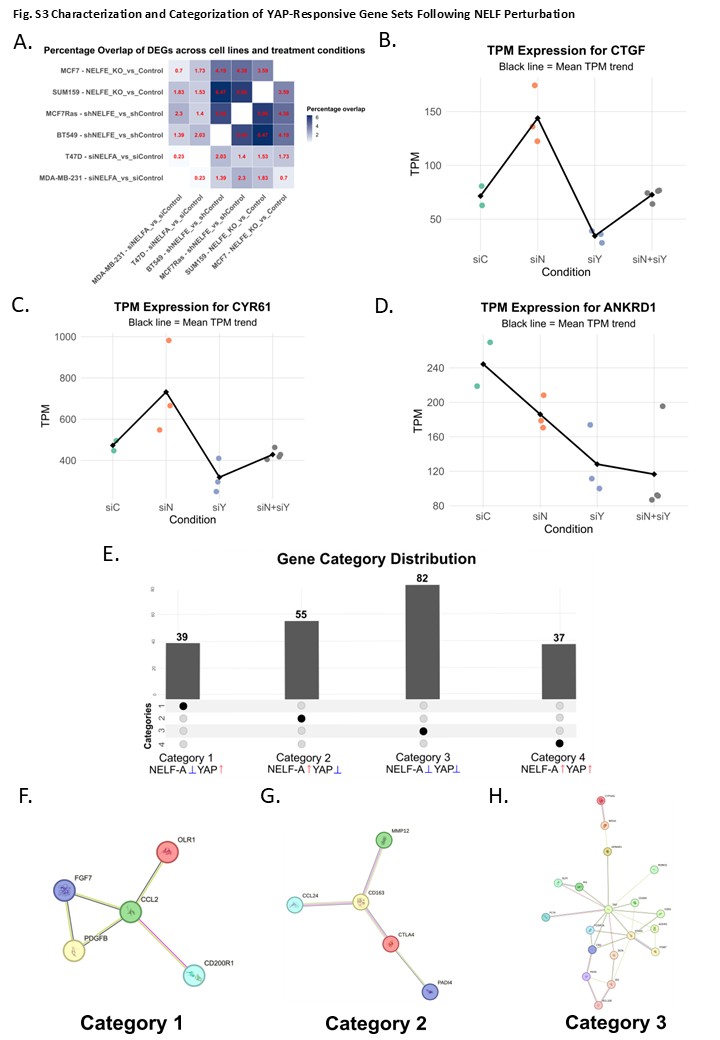

Supplement: Supplementary file 3 [file Image3.jpeg]

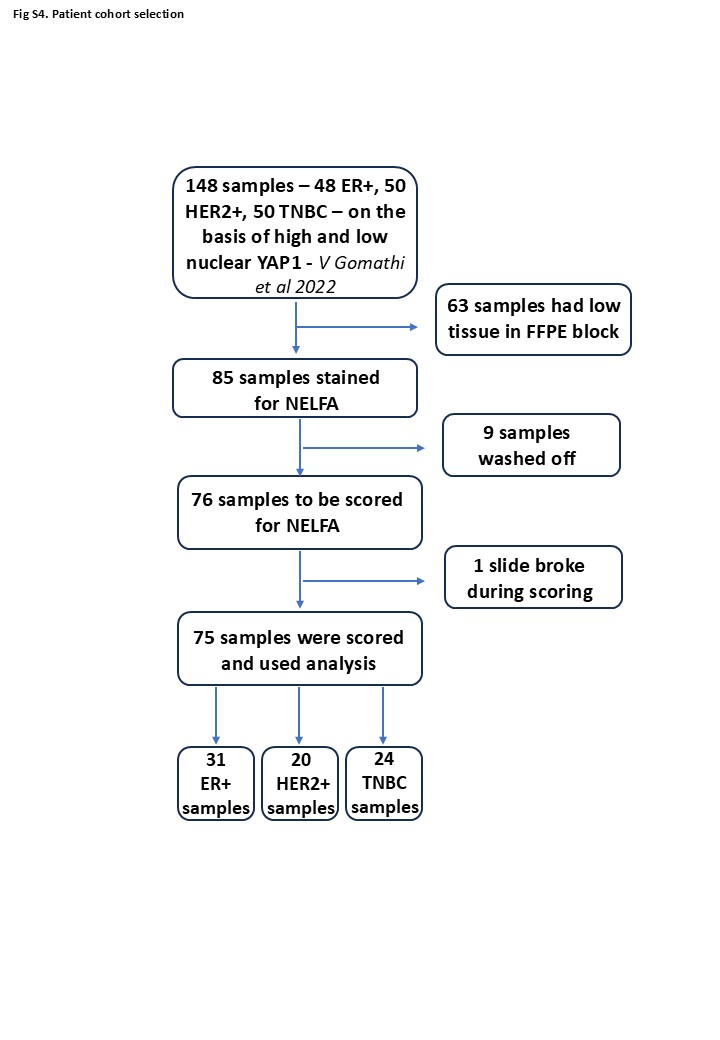

Supplement: Supplementary file 4 [file Image4.jpeg]

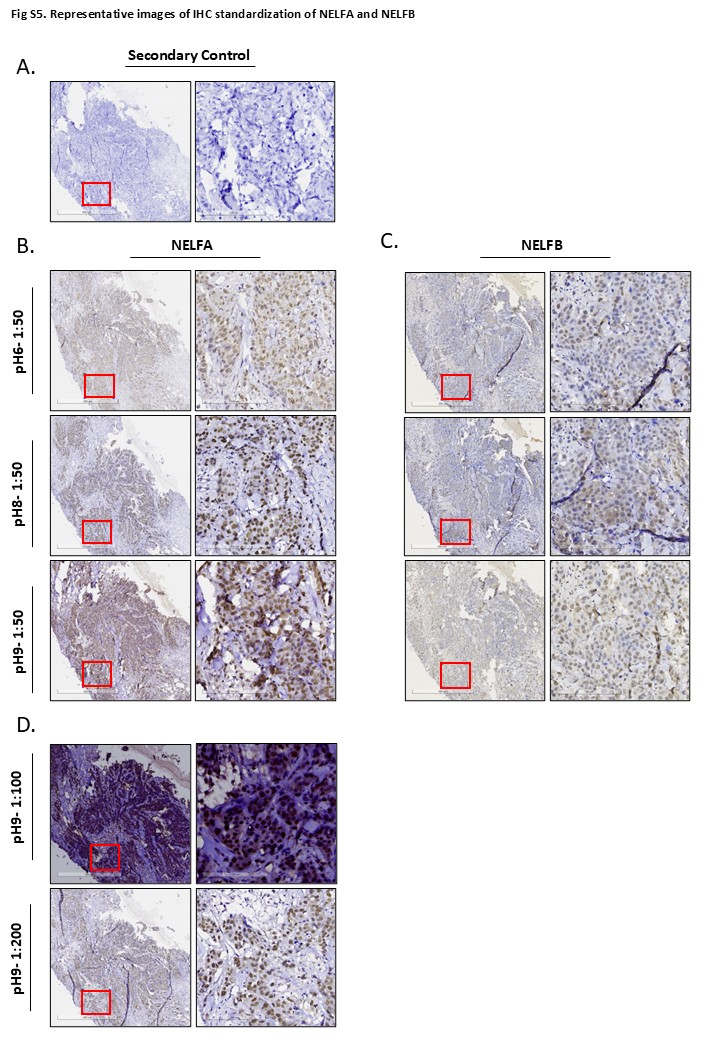

Supplement: Supplementary file 5 [file Image5.jpeg]

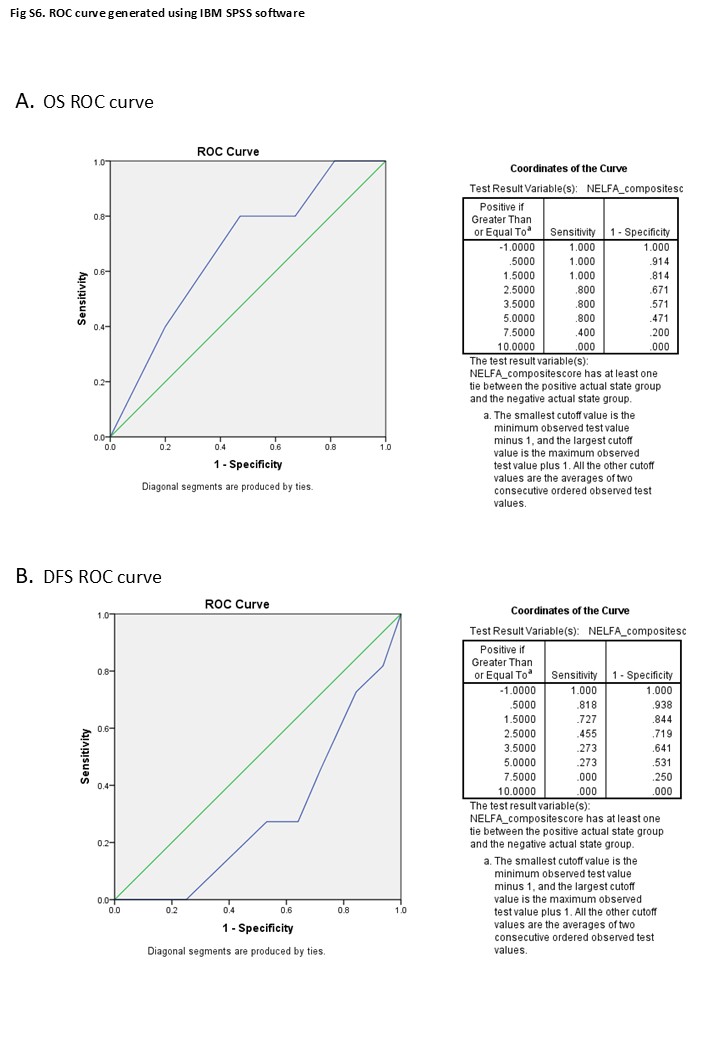

Supplement: Supplementary file 6 [file Image6.jpeg]

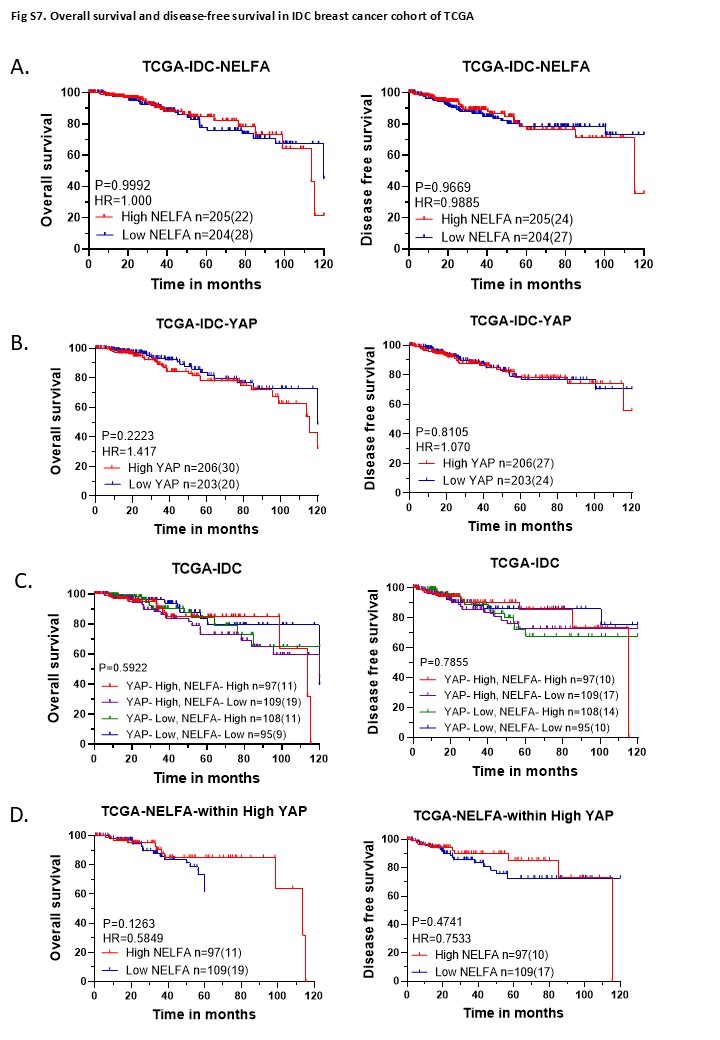

Supplement: Supplementary file 7 [file Image7.jpeg]

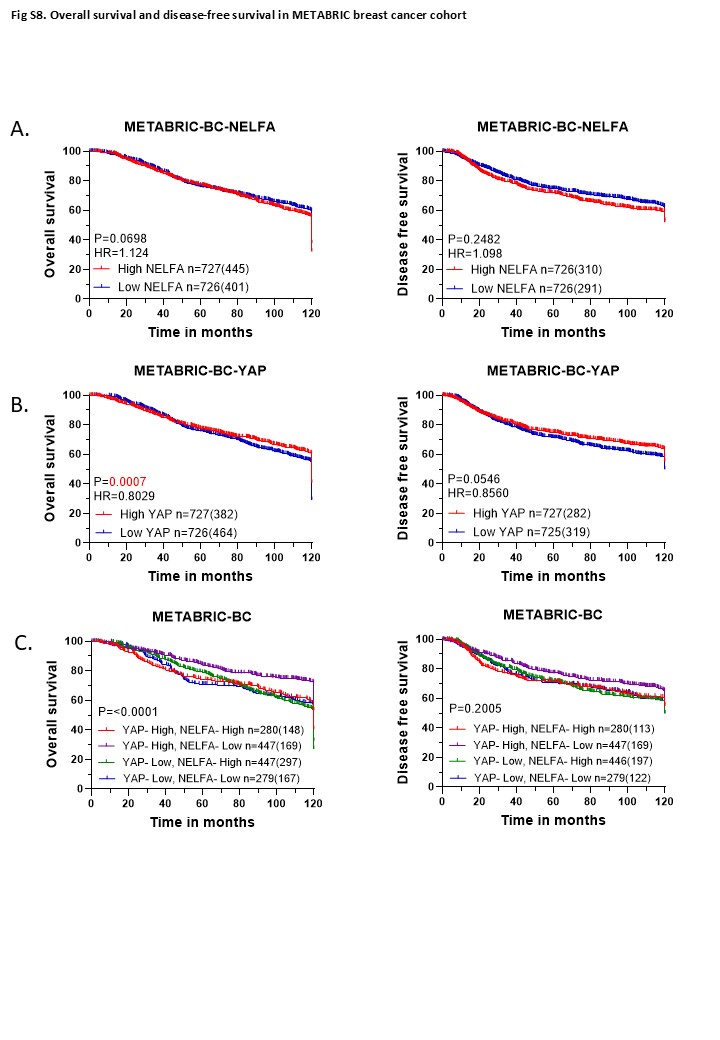

Supplement: Supplementary file 8 [file Image8.jpeg]
