# Supplementary material for: NELFA-mediated pausing restrains YAP transcription and context-dependent outcomes in breast cancer
Source: Front Oncol. 2026 Apr 22;16:1808415. doi: 10.3389/fonc.2026.1808415 (PMC13143776; doi:10.3389/fonc.2026.1808415)
Supplement: Supplementary file 9 [file DataSheet1.docx]

**Supplementary Methods:**

**CRISPR-Cas9 mediated knockout**: LentiCRISPRv2 plasmid (TLCV2) was a gift from Prof. Adam Karf at UNMC, Nebraska. The plasmid aliquot received was amplified by transformation in DH5α-competent *E. coli* cells and extracted using the QIAquick Plasmid Midi Kit (Qiagen, #12143) according to the manufacturer’s protocol.

Three gRNAs were designed against NELFA, out of which two targeted exon1 and one targeted exon2. We used Benchling, a cloud-based online platform, to design our gRNAs with high on and off-target scores. gRNAs were cloned into the LentiCRISPRv2 plasmid (TLCV2) using a restriction-based cloning method using the Lentiviral CRISPR toolbox protocol (GeCKO- Genome-Scale CRISPR Knock-out) by Zhang Lab. To generate CRISPR KO cells, we selected SKBR3 breast cancer cell lines. Lenti-X293T cells were co-transfected with gRNA against NELFA cloned TLCV2 and packaging vector pMD2 and psPAX2 using X-Fect transfection reagent (TAKARA, #631317). Viral supernatants were collected after 48 and 72 hrs and were stored at –80° C. SKBR3 cells were seeded in 6 x 6-well plates and were stably transduced with a 1:4:4 ratio using 8 μg/ml polybrene at 50% confluency. Stable cell lines were selected through one passage with 0.7ug/ml Puromycin and were induced with 1 µg/ml doxycycline for activation of CRISPR Cas9. Transduction was confirmed by GFP expression, and GFP-positive cells were FACS-sorted and maintained. Unfortunately, after multiple attempts, cells with stable GFP expression, i.e., those with a stable knockout of NELFA, were eliminated with subsequent passages.

**Table S1: List of Oligos for CRISPR knockdown**

| **Gene and Exon target** | **NELFA Exon 1-A** | **NELFA Exon 1-B** | **NELFA Exon 2** |
| --- | --- | --- | --- |
| **Forward** | **5'CACC**GACGGATGTTGTCGATGACCG 3' | **5'CACC**GCCGCATGGACGCCATCTTGG 3' | **5'CACC**GTCGGCGACCATGAGCACCC 3' |
| **Reverse** | **5'AAAC**CGGTCATCGACAACATCCGTC 3' | **5'AAAC**CCAAGATGGCGTCCATGCGGC 3' | **5'AAAC**GGGTGCTCATGGTCGCCGAC 3' |
| **On target score** | 71.0 | 66.1 | 60.5 |
| **Off target score** | 49.2 | 44.9 | 46.4 |
| **Cut position** | 5383 | 5272 | 22576 |

**Table S1: List of Oligos for CRISPR knockdown:**

Oligos were designed to target exon 1-A, exon 1-B, and exon 2 of NELFA gene. For each target, forward and reverse oligonucleotide sequences were designed. On-target scores indicate the predicted cut efficiency of each guide RNA, while off-target scores reflect the percentage of unintended genomic binding. The cut position denotes the predicted genomic cut site for each gRNA on the gene.

**Overlap of genes regulated by the PPP complex across studies**

Two other published studies report genes regulated by the PPP complex following knockdown of various NELF genes in breast cancer cell lines (Sun et al., 2010; Zhang et al., 2023). Sun et al looked at the effect of NELFA depletion in the T47D cell line, and Zhang et al looked at the effect of NELFE depletion, either with knockdown or knockout, in four different breast cancer cell lines: MCF7, MCF7Ras, SUM159, and BT549. We compared DEGs from these five diverse breast cancer cell lines with the DEGs derived after NELFA depletion in MDA-MB-231 in this study. To quantify transcriptional similarity between NELF-A or NELF-E perturbation conditions across cell lines, we calculated the pairwise percentage overlap of differentially expressed genes (DEGs) using the Jaccard index. All analyses were conducted using the readxl, stringr, and base R packages. The resulting matrix was used for heatmap visualization.

**STRINGDB Network Plots**

Protein-Protein Interaction (PPI) enrichment analysis for the significantly altered genes after perturbation of NELFA and YAP was conducted using the STRING-DB v12.0 database (<https://string-db.org>, Szklarczyk et al., 2023) to identify their functional connectivity. STRING-DB calculates a PPI enrichment p-value by comparing the observed number of edges with those expected in random networks of similar sizes, thereby estimating whether the submitted gene set forms a significantly interconnected network. In the STRING-DB network figure, edges represent functional associations supported by different evidence channels, as indicated by color: Green – neighborhood evidence, Red – gene fusion evidence, Blue – co-occurrence across genomes, Black – co-expression evidence, Pink – experimental interactions, Cyan – curated database links, Yellow – text-mining evidence.

**Metabric breast cancer cohort analysis**

Gene expression data for the METABRIC breast cancer cohort were obtained from the cBioPortal DataHub (https://www.cbioportal.org/). Expression profiling was performed using the Illumina HT-12 microarray platform, generating log2-normalized probe intensity values mapped to gene symbols, which were used for downstream analysis. Survival patient data was btained from the github link - <https://github.com/tmemklab/tcga_data_download/blob/main/run_tcga_biolinks.Rmd>. The dataset included patient sample information and associated clinical metadata.

Relevant information was curated using Microsoft Excel to isolate the desired subsets. All breast cancer samples, as described in[1] were selected for further analysis. For each patient sample, mRNA microarray gene expression, overall survival (OS), and disease-free survival (DFS) data (in months) were retrieved.

To categorize gene expression levels, the median of gene expression value was used as the cutoff to define high and low expression groups. Kaplan–Meier survival analyses were performed using GraphPad Prism (version 8) to generate survival curves based on OS and DFS.

**Supplementary Figure legends**

**Fig S1. Knockdown validation of PPP components**



**(A)** mRNA levels of MEPCE, NELFA and YAP following MEPCE knockdown and YAP induction in HEK293Ts (N=4) **(B)** Expression of HEXIM1, HEXIM2, NELFA, and YAP after YAP induction and HEXIM1/2 knockdown in HEK293Ts (N=4) **(C)** NELFA and YAP mRNA levels after NELFA knockdown and YAP overexpression in HEK293Ts (N=4) **(D)** Relative mRNA expression of NELFA, YAP, and **(E)** YAP target genes (Cyr61, CTGF, ANKRD1) in HEK293Ts and MDA-MB-231 cells (N=3). **(F)** Protein expression of NELFA and YAP confirmed by western blot in HEK293Ts and MDA-MB-231 (Mol.wt of GAPDH- 36 kDa, NELFA- 66 kDa, YAP- 65kDa) (N=2). **(G)** NELFA and YAP mRNA expression in MDA-MB-231 cells following NELFA and YAP knockdown (N=3). **(H)** Downregulation o

f NELFA and YAP protein levels validated by western blot using 20 µg whole cell lysate. Lanes: normal expression (-,-), siNELFA (+,-), siYAP (-,+), and combined siNELFA/siYAP (+,+). **(I)** Quantified protein level expression of NELFA and YAP in MDA-MB-231 using ImageJ software. All mRNA expression levels and protein level expressions were plotted using fold change values generated through a RT-qPCR and western blot and were normalized using GAPDH which served as an internal control. Significance was assessed using ordinary one-way ANOVA and multiple comparisons and the associated p-value is indicated in the bar plots where P<0.05 is considered significant when compared to control group for RT-qPCR.

**Fig S2.** **CRISPR workflow and representative images (A)** CRISPR Workflow Schematic. **(B)** Representative brightfield and GFP images of NELFA knockout SKBR3 cell lines.

**Fig S3**. **Cross-condition Transcriptomic Overlap and Network Architecture of NELFA–YAP Co-regulation**

**(A)** Percentage overlap of differentially expressed genes (DEGs) across NELFA/NELFE perturbation conditions in multiple human cell lines from Zhao et al 2023, Sun et al 2010, and our study [2,3] **(B), (C), (D)** TPM expression plots for canonical YAP targets CTGF, CYR61, and ANKRD1 for the three replicates for all four conditions. The black line represents the trend of mean TPM across replicates. **(E)** Upset Plot showing the distribution of genes across four regulatory categories. Genes were classified based on log2FC≥ 1 thresholds from siNELFA, siYAP, and double knockdown comparisons. Each bar represents the number of genes exclusively assigned to one of the four categories **(F), (G), (H)** STRINGDB protein–protein interaction (PPI) network for the three categories, with the PPI enrichment p-value = 7.06 × 10⁻⁵ for category one, PPI enrichment p-value = 1.67 × 10⁻⁴, for category two, PPI enrichment p-value = 1.3 × 10⁻¹² for category three.

**Fig S4**. **Patient cohort selection** from Prashanti Cancer Care Biobank and a Flowchart depicting number of patient samples selected, stained and scored for NELFA which were used for the survival analysis.

**Fig S5. Representative images of IHC standardization of NELFA and NELFB**:

Representative images of standardization for immunohistochemistry using NELFA and NELFB antibodies (give antibody details here): 3-micron Serial sections were taken for an IDC tumor sample to standardize the antibody dilutions, time, and retrieval buffer.

**(A)** Representative image of a secondary control with primary antibody. **(B)** NELFA representative images with antibody dilution of 1:50 and retrieval buffer pH 6, 8, and 9. **(C)** NELFB representative images with antibody dilution of 1:50 and retrieval buffer pH 6, 8, and 9. **(D)** NELFA representative images with retrieval buffer pH 9, antibody dilution of 1:100, 1:200.

**Fig S6.** ROC curve generated from composite score using IBM SPSS software

**Fig S7: Overall survival and disease-free survival of IDC breast cancer cohort from TCGA.**

**(A-D)** Kaplan-Meier (KM) plots are based on the NELFA and YAP expression cohort. Statistical significances are computed using the log-rank test (Mantel-Cox) in GraphPad Prism 8.0.1. For each KM Plot, the number of patients in each NELFA and YAP expression category is listed, along with the number of events in parentheses. **(A)** Overall and disease-free survival of the NELFA expressing IDC cohort **(B)** Overall and disease-free survival of the YAP expressing IDC cohort **(C)** Overall and disease-free survival of YAP+NELFA combination **(D)** Overall and disease free-survival of the NELFA expressing with high YAP IDC cohort.

**Fig S8: Overall and disease-free survival of METABRIC breast cancer cohort**

(A-C) Kaplan-Meier (KM) plots for IDC cohort from METABRIC, based on the NELFA and YAP expression cohort. Statistical significances are computed using the log-rank test (Mantel-Cox) in GraphPad Prism 8.0.1. For each KM Plot, the number of patients in each NELFA and YAP expression category is listed, along with the number of events in parentheses. **(A)** Overall and disease-free survival of breast cancer cohort based on the NELFA expression. **(B)** Overall and disease-free survival of the breast cancer cohort-based YAP expression **(C)** Overall and disease-free survival based on YAP and NELFA expression.

**Bibliography**

1. Venkatasubramanian, G., Kelkar, D. A., Mandal, S., Jolly, M. K. & Kulkarni, M. Analysis of Yes-Associated Protein-1 (YAP1) Target Gene Signature to Predict Progressive Breast Cancer. *J. Clin. Med.* **11**, 1947 (2022).

2. Sun, J. & Li, R. Human Negative Elongation Factor Activates Transcription and Regulates Alternative Transcription Initiation. *Journal of Biological Chemistry* **285**, 6443–6452 (2010).

3. Zhang, J. *et al.* Dependency of NELF-E-SLUG-KAT2B epigenetic axis in breast cancer carcinogenesis. *Nat. Commun.* **14**, 2439 (2023).
